# Green Synthesis, Characterization, Enzyme Inhibition, Antimicrobial Potential, and Cytotoxic Activity of Plant Mediated Silver Nanoparticle Using *Ricinus communis* Leaf and Root Extracts

**DOI:** 10.3390/biom11020206

**Published:** 2021-02-02

**Authors:** Anadil Gul, Fozia, Asmat Shaheen, Ijaz Ahmad, Baharullah Khattak, Munir Ahmad, Riaz Ullah, Ahmed Bari, Syed Saeed Ali, Abdulrahman Alobaid, Majid M. Asmari, Hafiz M. Mahmood

**Affiliations:** 1 Beijing Key Laboratory for Green Catalysis and Separation, Department of Chemistry and Chemical Engineering, Beijing University of Technology, 100 PingLeYuan, Chaoyang District, Beijing 100124, China; anadilgul97@gmail.com; 2 Biochemistry Department, KMU Institute of Medical Sciences, Kohat 26000, Pakistan; drfoziazeb@yahoo.com (F.); asmatshaheen@yahoo.com (A.S.); 3 Department of Chemistry, Kohat University of Science & Technology, Kohat 26000, Pakistan; 4 Department of Microbiology, Kohat University of Science & Technology, Kohat 26000, Pakistan; baharkk75@gmail.com; 5 Department of Physics, Hazara University, Mansehra 21330, Pakistan; munirkk87@gmail.com; 6 Department of Pharmacognosy (MAPPRC), College of Pharmacy, King Saud University, Riyadh 11451, Saudi Arabia; 7 Department of Pharmaceutical Chemistry, College of Pharmacy, King Saud University, Riyadh 11451, Saudi Arabi; abari@ksu.edu.sa (A.B.); sasyed@ksu.edu.sa (S.S.A.); aalobaid1@ksu.edu.sa (A.A.); masmary@windowslive.com (M.M.A.); 8 Department of Pharmacology, College of Pharmacy, King Saud University, Riyadh 11451, Saudi Arabia; harshad@ksu.edu.sa

**Keywords:** silver nanoparticles, *Ricinus communis*, antimicrobial activity, urease, xanthine oxidase, inhibition

## Abstract

The need of non-toxic synthesis protocols for nanoparticles arises developing interest in biogenic approaches. The present project was focused on cost effective, environment congenial synthesis of Ag nanoparticles and their biological applications. Leaf and root extracts of *Ricinus communis* were used as a reducing and stabilizing agent in synthesis process. A Proposed mechanism in published literature suggested that Indole-3-acetic acid, l-valine, triethyl citrate, and quercetin-3-0-*p*-d-glucopyranoside phytoconstituents of *Ricinus communis* act as reducing and capping agents. The synthesized Ag NPs were characterized with a help X-ray diffractometer, Transmission electron microscopy, UV-Vis spectrophotometry and Fourier Transform Infrared Spectroscopy (FTIR). The XRD results inveterate the synthesis of pure nano size crystalline silver particles. The FTIR data revealed the possible functional groups of biomolecules involved in bio reduction and capping for efficient stabilization of silver nanoparticles. TEM analysis confirmed the almost spherical morphology of synthesized particles with mean size 29 and 38 nm for R-Ag-NPs (root) and L-Ag-NPs (leaf), respectively. The stability of synthesized nanoparticles was examined against heat and pH. It was observed that synthesized nanoparticles were stable up to 100 °C temperature and also showed stability in neutral, basic and slightly acidic medium (pH 05–06) for several months while below pH 5 were unstable. The synthesized silver nanoparticles had promising inhibition efficiency in multiple applications, including as bactericidal/fungicidal agents and Urease/Xanthine oxidase enzymes inhibitors. The cytotoxicity of synthesized nanoparticles shows that the concentration under 20 μg/mL were biologically compatible.

## 1. Introduction

The Noble metals nanoparticles gained particular attention of the scientific community, because of its wide range application in various fields. Thus, Silver nano-particles (Ag-NPs) are of specific interest due to their exclusive chemical, physical, and biological properties as compared to bulk size [1]. Ag NPs have distinctive physical and chemical properties, for example, high thermal and electrical conductivity, surface enhanced Raman scattering, chemical stability, catalytic activity, nonlinear optical behavior, and antimicrobial effects [2]. Due to these properties, silver nanoparticles are broadly applied in many fields such as shampoos, soaps, detergents, cosmetics, toothpastes, electronic, optics, medical, and pharmaceutical products; hence, have direct effect on human health [3,4].

Synthesis of metal NPs is carried out by several chemical and physical protocols including solvo-thermal synthesis, laser ablation, chemical reduction, ion sputtering, and the sol–gel method, but the mentioned methods are quite expensive, time consuming, energy consuming, and environmentally toxic [5,6,7,8,9]. However, the need of environment friendly protocols for the production of NPs developed interest in biogenic approaches. Thus, synthesis of Ag NPs biogenically using micro-organism and plant extract have various significant of environmentally benign and its bio-medical applications. However, among biological methods the most adopted method is phytogenic synthesis Ag NPs because of its special advantages such as its wide distribution, easy availability, safe handling, and good source of metabolites [10]. Nanomaterial toxicity, mainly depend on its structural morphology like surface composition, charge and chemistry, shape, and particles size. Foe stability and enhance life span of metal NPs, the selection of synthesis protocol and non-toxic capping or stabilizing agents is an essential step [11]. To shun the adverse effects in medical application, there is an increasing demand for green synthesis via green nanotechnology. 

The nickel containing enzyme urease, present in plants, bacteria and few fungi were selected, to control some medical, agricultural, and environmental problems [12,13,14]. The function of Urease enzyme is catalysis of urea into ammonia with production of carbamate (intermediate); thus, increased the pH of the media. A large number of ureolytic bacteria are responsible for different infectious diseases in human being, such as *Helicobacter pylori* (*H. pylori*) present in the duodenum and stomach of human beings. The bacteria release Urease enzyme in stomach, which produce ammonia from urea; thus, reduce the pH of stomach and make the environment favorable for their survival, hence causes various gastric problems [15]. According to some literature, infection caused by *H. Pylori* was assumed to link with cardiac problems [7,8]. Furthermore, various ureolytic bacteria like *Proteus mirabilis* and *Klebsiella pneumonia* have also major role in formation of kidney stones and some urinary tract infections [16,17]. Xanthine oxidase, molybdenum-containing enzyme present in plant, bacteria, and animals. In humans its present in kidney liver, brain, plasma; however, liver and intestine have the highest concentration. It catalyzed the reaction of hypoxantaine into xanthine, and then xanthine into uric acid along with production of reactive oxygen (O^●^) and H_2_O_2_ which have adverse effect on body in high concentration. Thus, reactive species are associated with many diseases in human beings such as gout, Alzheimer’s, Parkinson’s, as well as related to aging [18]. Further, there is no specific literature available on these two-enzyme inhibitions with silver nanoparticles synthesized by *Ricinus communis* leaf as well as root extract.

In the present study *Ricinus communis* plant (Root and leaf) extract was used as a reducing and stabilizing agent for synthesis of Ag NPs. *Ricinus communis* (commonly called as Castor Bean) is an annual medicinal plant of Pakistan which contain bio reductants and stabilizers such as polysaccharides, flavonoids, alkaloids, tannins, phenolics, saponins, and steroids [19]. Further, structural and morphological study of synthesized Ag NPs was carried out by using various techniques such as X-ray diffractometer, Transmission electron microscopy, UV-Vis spectrophotometry and Fourier Transform Infrared Spectroscopy (FTIR). Moreover, the inhibition activity of synthesized NPs was checked against Gram-negative and Gram-positive bacteria, two different fungal strains, also studied enzyme inhibition. Furthermore, evaluated its cytotoxicity by using hemolysis assay to determine its biocompatibility for practical application.

## 2. Materials and Methods

### 2.1. Plants Collection

*Ricinus communis* fresh plants were collected from the region of Karak, KPK, Pakistan. The plant was authenticated from Department of Botany, Kohat University of Science & Technology. The plant was dried in shade for several days than the leaf and roots were washed with double distilled water many time for removal of dust particles. The cleaned plant parts were again dried and separately dipped in methanol for a week. After one week, filtered the mixture (remove plant parts) and the filtrate was subjected to rotary evaporator to remove solvent thus methanolic extract was obtained. Then, 100 mg of methanolic extract was dissolved in 100 mL distilled water to prepared 1% plant extract, the prepared solution was filtered, and the filtrate was used for reduction of silver ions Ag^+^ to Ag^0^.

### 2.2. Green Synthesis of Silver Nanoparticles

The green protocol was used for synthesis of Ag NPs as reported in literature [20]. *Ricinus communis* leaf and root extract was used as a capping and reducing agents. A stock solution (1 mM) silver nitrate was prepared, the metal ligands with different amount were mixed with plant extract (1%) solution in the ratio: 1:1, 1:2, 1:3, 1:4, 1:5, 1:6, 1:7, 1:8, 1:9, 1:10 mL (taking plant solution constant), and also invers of this took 10 mL constant AgNO_3_ solution and took plant solution variable from 1 to 10 mL. The mixtures were than stirred for 4 h. After completion of reaction the change in color from transparent to brown was observed, centrifuged the samples and collected for onward application. Than the reaction mixture, was examined by UV-Visible spectrophotometer. The synthesized sample were mentioned as R-Ag-NPs and L-Ag-NPs for root and leaf extract synthesized Silver NPs, respectively.

### 2.3. Characterization

UV-Vis spectroscopy was performed in the 200–700 nm for initial synthesis conformation of nanoparticles with reference to distal water by using UV-Vis spectrophotometer (Shimadzu-UV-1800, Kyoto, Japan). Fourier Transform Infrared Spectrophotometer (Billerica, MA, USA) was used for examination of fictional groups of the biomolecules present in plant extract used for R-Ag-NPs and L-Ag-NPs. Transmission electron microscopy was performed for to studies the structural morphology of synthesized nanoparticles and to calculate particle size by using Transmission electron microscope JEM-2100 (Jeol, Tokyo, Japan). The particle size was calculated from TEM micrograph by using image-j software (1.51j8) [21]. X-ray diffractometer (Brikar-D8 advance, Bruker, Billerica, MA, USA) was used to examine the crystallinity of and particle size of synthesized NPs in the range of 30–80 2 theta, using irradiation Ka Cu (λ = 0.1541 nm). Scherer’s equation was used to calculate crystallite size from XRD patterns [22].

### 2.4. Biological Evaluation Methods

#### 2.4.1. Xanthine Oxidase Assay

The test sample (Ag NPs) inhibitory potential against xanthine oxidase was determined by the hydroxylation reaction of xanthine (substrate) in which uric acid (colorless) formed as an end product. The uric acid shows adsorption at 296 nm. The reaction mixture, which was used in this protocol, consists of sample, phosphate buffer, xanthine, and xanthine oxidase enzyme. First 1 mmol/L solution of pure sample was prepared and then 10 μL of this was dissolved in Dimethyl sulfoxide (DMSO). 0.003 units of XO enzyme were dissolved in 20 μL of buffer (phosphate buffer). 20 μL of xanthine (0.1 mmol L^−1^) was used as substrate. When XO was added then this mixture was incubated for 10 min at room temperature. After incubation the mixture was first analyzed in the UV region (λ max 295 nm). Then substrate was added to the mixture, and continues reading for 15 min at an interval of 1 min was observed (Spectra MAX-340, Molecular Devices, San Jose, CA, USA). The % inhibition of test sample (AgNPs) was calculated by using the formulas:

(%) Inhibition = 100 − [(OD test compound/OD control) × 100](1)

OD = A/L(2)

where A is absorbance and L is the thickness of the sample and then absorption is calculated by the following formula:

A = aλ × b × c(3)

where aλ is absorptivity coefficient which depend upon λ, b is path length and c is concentration of analyte. By using EZ-Fit windows-based software version 5.03 (Perrella Scientific Inc. Amherst, MA, USA) IC_50_ values of the compounds was calculated. Allopurinol was used as positive control (standard). The inhibitory activities of the test samples were then compared with the standard. The reaction for each compound was performed in triplicate [23].

#### 2.4.2. Urease Assay

Jack bean urease (25 μL), 55 μL of buffers containing 100 mM urea and test sample (5 μL) were incubated at 30 °C for 15 min in 96-well plates.

Indophenol method was used for estimation of ammonia production to determine the Urease activity. Briefly, 45 μL each phenol reagent (1% *w*/*v* phenol and 0.005% *w*/*v* sodium nitroprusside) and 70 μL of alkali reagent (0.5% *w*/*v* NaOH and 0.1% active chloride NaOCl) were added to each well. After 50 mints micro plate reader was used for the increasing absorbance at 630 nm. The experiment was accomplished in triplicate at 200 μL final volume. Per min change in absorbance were sorted out by using molecular Device, USA, Soft Max Pro 6 software. The assays were executed at pH 6.8. Percentage inhibitions were calculated from the formula:

100 − (OD_testwell_/OD_control_) × 100 (4)

Thiourea was used as a control [24].

#### 2.4.3. Antimicrobial Assessment

Four bacterial strains *Streptococcus pneumoniae*, *Klebsiella pneumoniae*, *Escherichia coli*, *Staphylococcus aureus*, and two fungal strains *Alternaria alternate* and *Aspergillus niger* were used. These organisms were selected because they are common pathogens which cause various intestinal diseases, and skin and blood infections. All these organisms were taken from Department of Microbiology Kohat University of Science & Technology, Kohat, Pakistan. The anti-bacterial and anti-fungal study was performed by using Ager well diffusion method reported in literature [25]. Briefly, first inoculated SDA ager (25 mL) on Petri plate after solidification of ager the microorganism strain was distributed by using swab (sterile). Onward, with the help of plastic borer, made wells then put 1 mL sample Ag-NPs (5 mg/L) in the well, also used standard in one well and incubated the plats after incubation measured the zone of inhibition and compared with standard. 

#### 2.4.4. Cytotoxicity of Ag Nanoparticles Hemolytic Potentiality Test

Hemolysis is the damaging process of RBC membrane, and thus cause the release of hemoglobin in to plasma [26]. The hemolysis activity of Ag nanoparticles was examined to check their biocompatibility, by using Miki et al. method with little modifications [27]. Firstly, 9 mL of blood sample was collected from sheep, to inhibit the coagulation mixed it with sodium citrate (1 mL/3.8%). The obtained sample was centrifuged for 5 min at 3000 rpm, the supernatant was removed and the pellet of RBC was dispersed in phosphate buffer saline (PBS/10 mL). For complete removal of buffy coat of RBS the same processes was repeated 3 times. At last, the RBS were dispersed in PBS to obtained uniform suspension of cells. Different concentration (2, 4, 8, 12, and 20 μg/mL) of Ag NPs were took in test tubes than added 2 mL RBC suspension and were shake gently to mix the nanoparticles and cells. Afterward, the tubes were incubated for 90 min at 37 °C and for positive and negative control 2 mL of RBC suspension was added to triton X-100 and PBS having pH 7.4, respectively. After 90 min incubation the sample were centrifuged for 5 min at 3000 rpm and the supernatant was checked by UV-vis spectrophotometer at λ_max_ 540 nm while PBS was used as a blank solution. The % hemolytic index was calculated by using the following formula [28].

Hemolysis percentage = (OD_test sample_ − OD_negative control_)/(OD_positive control_ − OD_negative control_) × 100 (5)

The optical density value of positive and negative controls was found to be 1.301 and 0.005, respectively.

## 3. Results and Discussion

**Figure 1:**
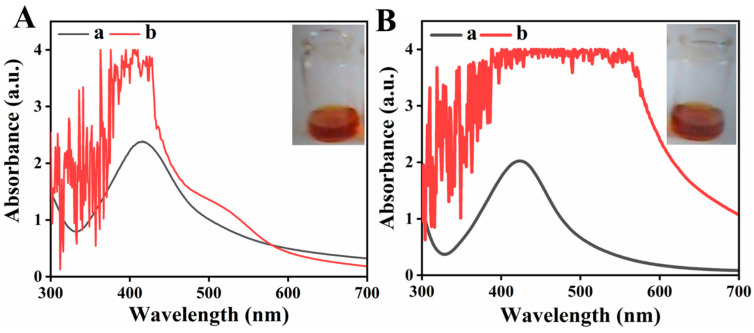
UV-Vis spectra of (**A**) Root (**B**) Leaf where (a) plant extract and (b) Corresponding Silver nanoparticles.

*Ricinus communis* root and leaf extracts were used for reduction of AgNO_3_, and were also used as stabilizer. The optimized ratio in case of root extract was 1:4 (extract: AgNO_3_) while in case of leaf extract was 1:5. Initially the synthesis of Ag nanoparticles was confirmed from the change in color of reaction mixture after reduction of Ag, as shown in Figure 1. It is well-known that due to SPR (Surface Plasmon resonance) the appearance of Ag NPs in water is brownish color, which is initial synthesis indication of Ag NPs. The observed results were similar to previous reports, in which Ag NPs were synthesized by using leaf extract of various plants and aqueous Silver nitrate solution (10^−3^ M) change its color from transparent to brown after extract addition [29].

### 3.1. UV-Vis Spectrophotometry

UV-Vis Spectroscopy is an essential analytical technique used for the synthesis confirmation of metal NPs, provide SPR band for the metal. This technique could also be used for shape and size examination of nanoparticles in aqueous suspension [30]. In metal nanoparticles, the conduction (CB) and valance band (VB) of metal NPs such is Ag silver lie too close to each other thus electron move freely. When the collective oscillation frequency of electrons become equal with the incoming wave (UV light) frequency than strong absorption takes place, which is responsible for production surface plasmon resonance (SPR) absorption band. The absorption band is size, surrounding environment and di-electric medium depended [31,32,33,34]. Figure 1A,B reveal the characteristic surface Plasmon resonance absorption band at 418 nm and 424 nm for silver nanoparticles synthesized by using root and leaf extracts, respectively. Figure 1A,B also reveal the U.V Visible spectra of *Ricinus communis* root and leaf extracts so it was confirmed that only colloidal suspensions of silver after reduction shows strong absorbance in the range of 400–450 nm. The obtained results are similar to reported literature in which reduction of silver nanoparticles using *Spirulina platensis* was carried out and UV-visible absorbance spectra shows the SPR band for silver occurred in a range 400–480 nm [35].

### 3.2. X-ray Diffraction Analysis (XRD)

**Figure 2:**
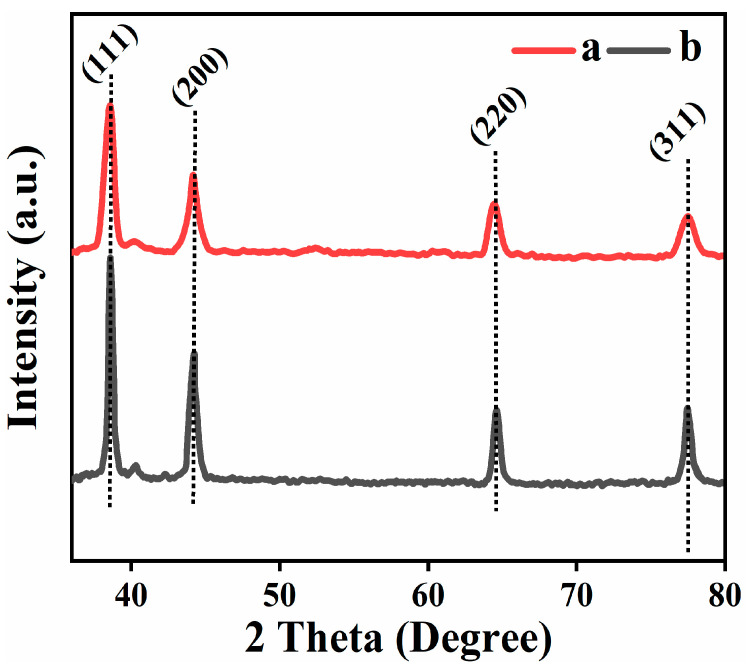
XRD patterns of synthesized NPs (a) R-Ag-Nps and (b) L-Ag-Nps.

The crystallinity of synthesized composites was investigated by using X-ray crystallography in the range of 35–80-degree 2θ. Figure 2 shows the XRD patterns of both the samples confirmed the crystal structure of Ag-Nps (face canter cubic) having 4 characteristic peaks at 2 θ position 38.5, 44.2, 64.4, and 77.5° correspond to *hkl* planes (*111*), (*200*), (*220*) and (*311*), respectively (JCPD, NO. 04-0783) [36]. The particle size are 17 nm and 22 nm for R-Ag-Nps and L-Ag-Nps, respectively, calculated by using Scherrer’s equation [37]: D = 0.9 λ.β.cos θ where D is size of crystallite, λ correspond to X-rays wavelength used 1.5406 Å, β is FWHM (full width half maximum) and θ correspond to Bragg’s angle.

### 3.3. Fourier Transform Infrared Spectroscopy (FTIR)

**Figure 3:**
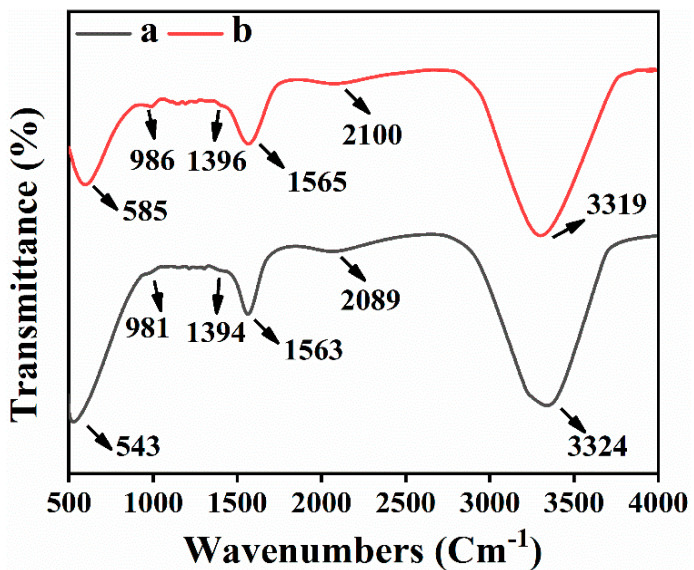
FTIR spectra of (a) leaf extract of *R. communis* (b) Silver nanoparticles synthesized by using 1% leaf extract of *R. communis*.

FTIR analysis was executed to examine the bio-molecules used as a stabilizing and reducing agents for R-Ag-NPs and L-Ag-NPs synthesis. Figure 3 (curves a, b) shows IR spectrum of R-Ag-NPs and L-Ag-NPs the absorption bands around 543, 981, 1394, 1563, and 2089 cm^−1^ revealed the presence of alkyl halides, proteins, phenolic, and aromatic compounds [38]. Among them, the most characteristic peak was the peak at 3379.0 cm^−1^ corresponds to O-H stretching, inter-molecular hydrogen bonding [39]. The absorbance peak located at around 981 and 996 cm^−1^ can be assigned is the C-N stretching vibrations of amine. The peak around 1396 and 1394 cm^−1^ correspond to N-H stretching vibration due to amide linkage (proteins), specially play important role in reducing and capping of Ag nanoparticles similar to many reported literature [40,41]. The band around 2100 and 2089 cm^−1^ were associated to alkyne moiety present in extract phyto-constituents. The peak around 543 and 585cm^−1^ can be assigned to –CH=CH (cis) vibration; however, the band around 600 cm^−1^ can also be correlated with metal oxygen bond [42].

### 3.4. Transmission Electron Microscopy

**Figure 4:**
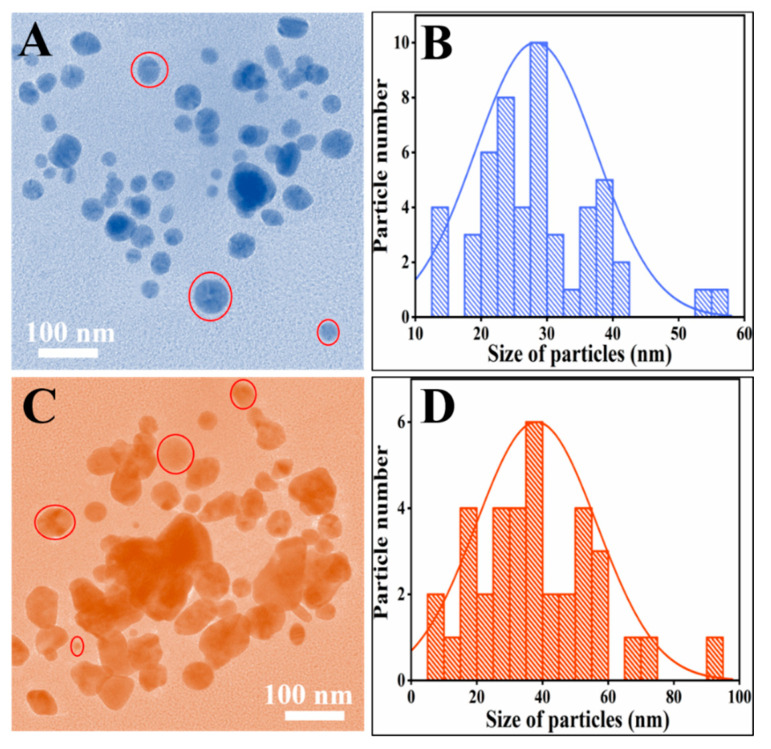
TEM micrographs of (**A**) R-Ag-NPs and (**B**) L-Ag-NPs and their relative particles size distribution histogram (**C**,**D**).

The size of synthesized green NPs were examined by Transmission electron microscopy (TEM). Figure 4 show the TEM micrographs of R-AG-NPs (Figure 4A,B) and L-AG-NPs (Figure 4C,D), it can be seen from the micrographs that the almost the particles have spherical morphology. Figure 4B,D demonstrate the histogram of particles size distribution calculated by image-j software [21]. Figure 4A revealed that the particles are spherical and dispersed and histogram (Figure 4B) showed that that the particles size range from 13 to 56 nm while the mean particle size 29 nm. However, Figure 4C shows that the particles shape is almost spherical with some aggregation also, the range of particles size was from 10 to 100 nm while the mean particle size is 37 nm.

### 3.5. Heat and pH Stability of Synthesized Nps 

**Figure 5:**
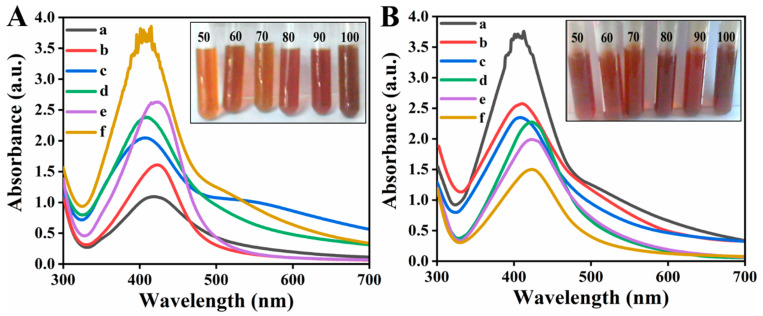
UV spectra of (**A**) L-Ag-Nps (**B**) R-Ag-Nps at different temperature (a) 50 °C, (b) 60 °C, (c) 70 °C, (d) 80 °C, (e) 90 °C and (f) 100 °C.

Heat stability of R-Ag-NPs and L-Ag-NPs was checked by heating at various temperatures, i.e., 50, 60, 70, 80, 90, and 100 °C for 30 min as shown in Figure 5. It was found that in both cases the synthesized Ag NPs were stable up to 100 °C. Figure 5A,B shows the absorption peaks 410 nm and 407 nm at 100 °C temperature for L-Ag-NPs and R-Ag-NPs, respectively. The observed peaks are the characteristic plasmon band for Ag NPs [43]. It can be seen from Figure 5 that increase in the intensity of peak occur with increasing temperature, it could be correlated with increase in particles size. Furthermore, the shift in peak toward lower wavelength from 424 and 418 nm (50 °C) to 410 and 407 (100 °C) occurred with increasing temperature, this blue shift occurred due to the change in particle shape and size [44].

**Figure 6:**
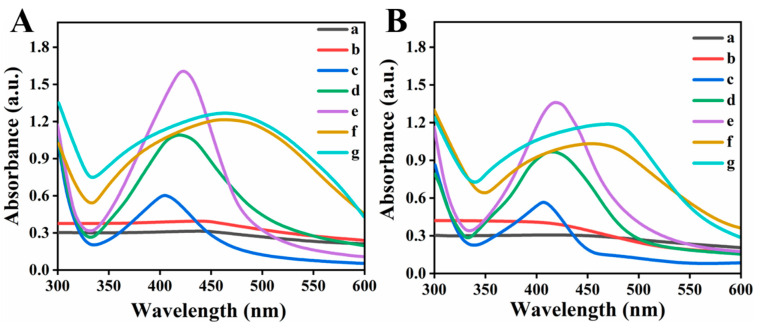
UV spectra of (**A**) R-Ag-NPs and (**B**) L-Ag-NPs stability at different pH (a) 1–2, (b) 3–4, (c) 5–6, (d) 7–8, (e) 9–10, (f) 11–12 and (g) 13–14.

The pH stability of synthesized silver nanoparticles L-Ag-Nps and R-Ag-Nps had been examined by changing the pH of the solution (Figure 6A,B). The results revealed that the synthesized particles were stable in neutral (pH 7–8, curve d, Figure 6) and basic medium (pH 9–14, curves e, f, g, Figure 6) for several months while in acidic medium (pH 1–4, curves a, b, Figure 6) they were stable up to 05–06 pH and below pH 5 were unstable, similar to reported literature [45]. However, in basic media broadness and batho-chromic shift occur in peak position; this change can be correlated to the change in particles size with respect to pH change. The broadness occurs because of dominant contribution of higher order electron-oscillation, thus suggested the increase in particle size [45]. The band broadening is obvious due to the dominate contributions from higher order electron oscillations, specially seem in case of high basic condition (pH 11–14), correspond to huge increase in particle size (according to Mie theory). While in case of acid media (4–5) hypso-chromic effect occur and intensity become decreased which suggested decrease in particle size [46]. 

### 3.6. Biological Activity Analysis

#### 3.6.1. Enzymes Inhibition 

**Figure 7:**
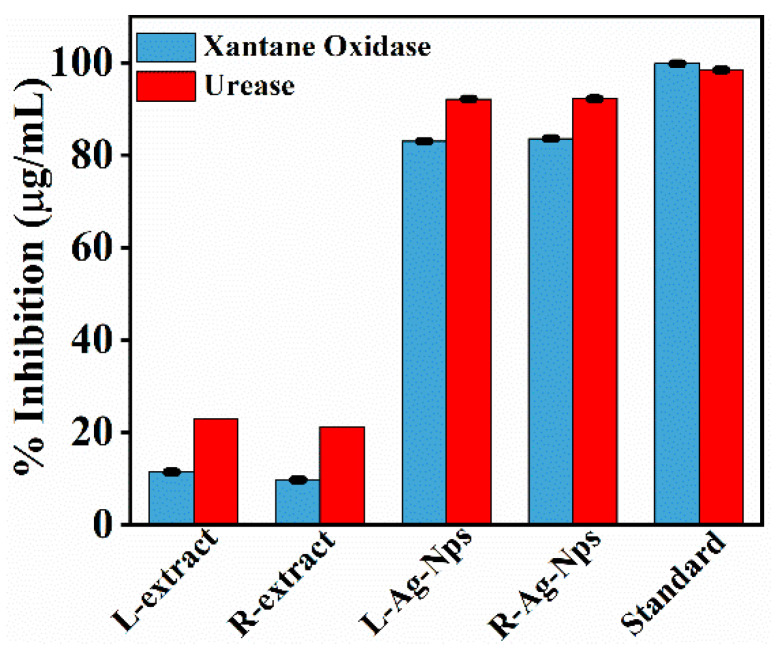
Enzyme inhibition of plant extract and plant extract stabilized Ag NPs. (Standard) Allopurinol and Thiourea.

The synthesized particles R-Ag-NPs, L-Ag-NPs and plant (*Ricinus communis)* extract were examined for inhibition of two enzymes Xanthine oxidase and Urease. The Inhibition investigation was executed to explore the inhibition efficacy of synthesized Silver NPs. The obtained results demonstrated that the synthesized nanoparticles (R-Ag-NPs and L-Ag-NPs) showed an excellent efficacy against these enzymes as shown in Figure 7. It was found that R-Ag-NPs were more active against Urease, which have 36.81 ± 0.05 µg/mL IC_50_ value and 94.2% inhibition activity, while in case of Xanthine Oxidase the IC_50_ value was 3.60 ± 0.04 with 83.6% inhibition. However, the L-Ag-NPs showed 92.1% inhibition activity against Urease with IC_50_ values 38.15 ± 0.02, while in case of Xanthine Oxidase the % inhibition was 83% with IC_50_ value 3.8 ± 0.03. Hence, it was concluded that the synthesized Ag NPs (R-Ag-NPs and L-Ag-NPs) have an excellent inhibition activity against the two enzymes Urease then Xanthine Oxidase.

#### 3.6.2. Antibacterial Evaluations 

**Figure 8:**
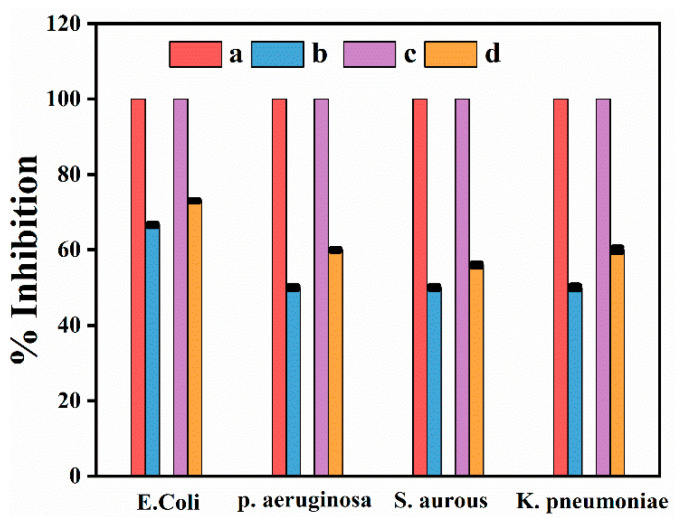
Antibacterial activities of root and leaf stabilized Ag NPs. (a) standard, (b) L-Ag-NPs, (c) Standard (Levofloxacin) and (d) R-Ag-NPs.

The antibacterial assessment of newly synthesized Ag NPs was carried out against four different strains of bacteria i.e., *E. coli*, *Pseudomonas aeruginosa* *Staphylococcus aureus*, and *Klebsiella pneumoniae* show in Figure 8. It was observed that R-Ag-NPs show highest activity 73% against *E. coli* and 60%, 56%, and 60% against *P. aeruginosa*, *S. aurous*, and *K. pneumonia*, respectively. Furthermore, L-Ag-NPs show highest activity 66% against *E. coli* while 50, 50, and 49 against *P. aeruginosa*, *S. aurous,* and *K. pneumonia*, respectively. The activity of R-Ag-NPs is higher than L-Ag-NPs might be due to difference in shape and particles size [47].

The results concluded that newly synthesized Ag NPs revealed an excellent efficiency against Gram-negative and Gram-positive bacteria *E. coli*, *P. aeruginosa*, *K. pneumonia*, and *S. Aurous*, respectively. Further, the extract solution of both leaf and root had no activity, used in the same concentration as used in the synthesis of nanoparticles.

#### 3.6.3. Antifungal Evaluations 

**Figure 9:**
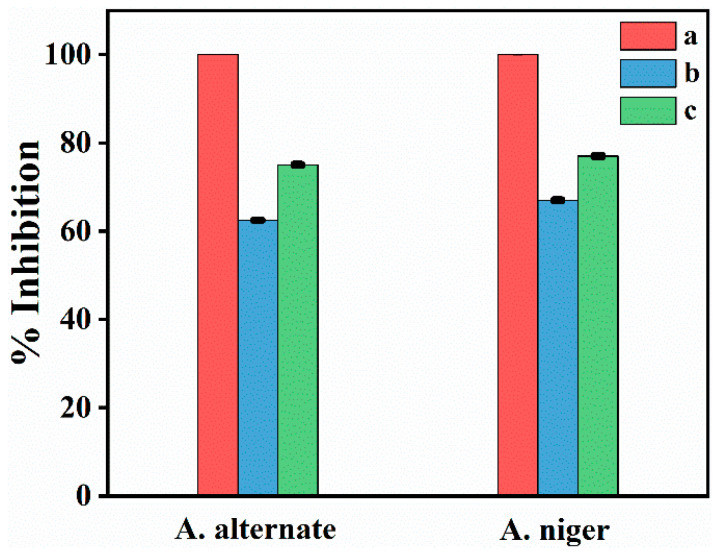
Antifungal activities of (a) Standard (Levofloxacin), (b) L-Ag-NPs and (c) R-Ag-NPs.

The efficacy of synthesized nanoparticles was examined against two fungal strains *Aspergillus niger* and *Alternaria alternate* shown in Figure 9. The results demonstrated that R-Ag-NPs show 77% and 75% inhibition efficiency against *Aspergillus niger* and *Alternaria alternate*, respectively. However, the L-Ag-NPs show 67 and 63% inhibition efficiency against *Aspergillus niger* and *Alternaria alternate*, respectively. The activity of R-Ag-NPs were higher than L-Ag-NPs due to shape and particle size they were more reactive, according to Yaohua Dong et al. the activity of smaller silver nanoparticles are higher than larger particles [47]. Further, the extract solution of both leaf and root had negligible activity, used in the same concentration as used in the synthesis of nanoparticles.

#### 3.6.4. Cytotoxicity Analysis of Ag-NPs (Hemolytic Assay)

**Figure 10:**
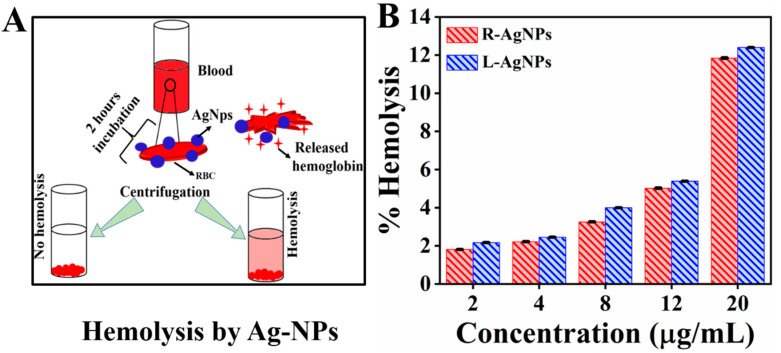
(**A**). Schematic presentation of hemolysis process (**B**). % hemolysis of synthesized nanoparticles at different concentration. The positive and negative control showed 100 (±0.001) and 0 (±0.001) % hemolysis.

Silver nanoparticles are increasingly use in the field of biomedical due to its incredible antimicrobial activity. Therefore, along with the potential application of silver NPs in biomedical field, the knowledge about its biocompatibility also gained much attention of scientific community. Blood is the major carrier for any foreign substance (nanoparticles), carried them to organs, tissues and cells, specifically erythrocytes [48]. Thus, the biocompatibility of Ag NPs with blood is consider more important for its practical application in biomedical field. Figure 10 show the % hemolysis of Ag nanoparticles at different concentration (2, 4, 8, 12, and 20 μg/mL) and also its schematic representation, it can be seen that the hemolysis activity increased with increasing concentration of Ag nanoparticles. The results shows highest hemolysis at 20 μg/mL in both cases R-Ag NPs (12.4%) and L-Ag NPs (11.8%), which are almost in good agreement with reported literature [49]. Although, the difference b/w two sample is less but overall hemolysis % by R-Ag NPs was greater than L-Ag NPs. Thus, this increase can be correlated with size of nanoparticles, as the hemolysis is size depended. Chen et al. studied the hemolysis activity of three different size Ag nanoparticles 15, 50, and 100 nm, they concluded that the small size nanoparticles (15 nm) showed higher hemolysis activity ~60% than the larger size particles. Thus, the present nanoparticles show good biocompatibility up to 12 μg/mL (R-Ag NPs: 5.3%, L-Ag NPs: 5.01%) and beyond this limit the % hemolysis exceeded the permissible limit, as 5% hemolysis is permissible according to ASTM-E252408 for biomaterials [50].

### 3.7. Proposed Mechanism for Ag Nanoparticles 

**Scheme 1:**
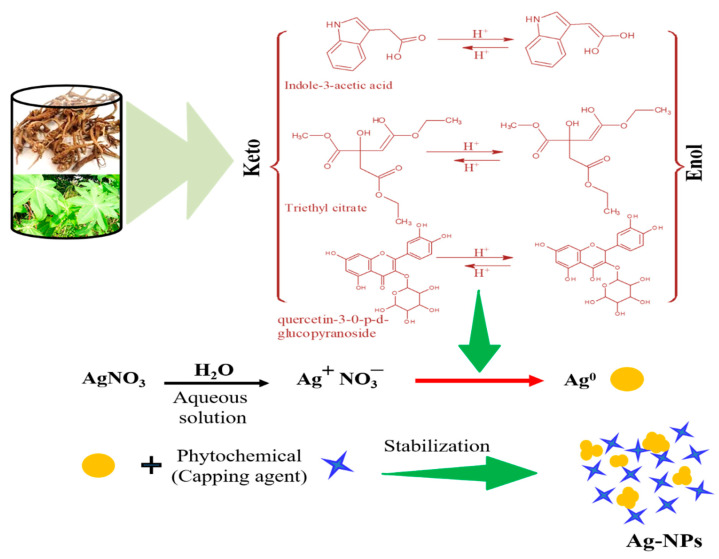
Proposed mechanism for Ag nanoparticles synthesis.

According to reported literature [51,52,53,54], the roots and leaves of *Ricinus communis* contained various phenolic and flavonoid compounds such as *n*-haxadecanoic acid, octadecanoic acid, 1-hexadecanol. 2-methyl, l-valine, triethyl citrate, diethyl phthalate, gallic acid, kaempferol-3-*o*-b-d-xylopyranoside, kaempferol-3-*o*-b-d-xylopyranoside, and quercetin-3-0-*p*-d-glucopyranoside; however, the major consistent of root extract is Indole-3-acetic acid. Although, the exit mechanism of metal nanoparticle by using plant extract is unknown due to the complex chemicals composition of plants (extract); however, on the base of above observation we can proposed general mechanism for synthesis of silver nanoparticles with three compounds as shown in Scheme 1. The Indole-3-actetic acid (root) keto form convert into enol form, with liberation of hydrogen (reactive); however, due to two hydroxyl groups on same carbon the enol form was unstable and convert back into keto form. Thus, the liberated reactive hydrogen convert Ag^+^ into Ag^0^, which combine with each other to form Ag nanoparticles. Similarly, in case of leaf extract, triethyl citrate, flavonoid (quercetin-3-0-*p*-d-glucopyranoside) was considered as reducing agents. The phytochemical such as phenolic compounds and amino acid (1-valine) also play important role in the stability of metal nanoparticles [55,56]; thus, stabilized the Ag nanoparticles.

## 4. Conclusions

The current research work concluded that *Ricinus communis* has various compounds in which mainly Indole-3-acetic acid, l-valine, triethyl citrate, quercetin-3-0-*p*-d-glucopyranoside act as reducing and capping agents in the synthesis of silver nanoparticles. The mean particle size of R-Ag-NPs (Root) was smaller 29 nm than L-Ag-NPs (leaf) 37 nm. Although, the change in size and shape of nanoparticles occurred by changing the temperature and pH but overall they were stable at high heat (100 °C) and neutral, basic (upto pH 10), and slightly acidic medium (5–6) below this pH they were unstable. Further, the synthesized silver nanoparticles were active against fungal strains, Gram-positive and Gram-negative bacterial strains and also showed potent inhibition against Xanthine oxidase and Urease enzymes. The R-Ag-NPs were more potent than L-Ag-NPs against various microorganism inhibition. However, the biocompatibility (hemolysis assay) of L-Ag-NPs were higher than R-Ag-NPs, thus up to 12 μg/mL we can use Ag nanoparticles practically in biomedical field.

## Data Availability

All the data incorporated in the manuscript.
